# Comparison of six fit algorithms for the intra-voxel incoherent motion model of diffusion-weighted magnetic resonance imaging data of pancreatic cancer patients

**DOI:** 10.1371/journal.pone.0194590

**Published:** 2018-04-04

**Authors:** Oliver J. Gurney-Champion, Remy Klaassen, Martijn Froeling, Sebastiano Barbieri, Jaap Stoker, Marc R. W. Engelbrecht, Johanna W. Wilmink, Marc G. Besselink, Arjan Bel, Hanneke W. M. van Laarhoven, Aart J. Nederveen

**Affiliations:** 1 Joint Department of Physics, The Institute of Cancer Research and The Royal Marsden NHS Foundation Trust, London, United Kingdom; 2 Department of Medical Oncology, Academic Medical Center, Amsterdam, Netherlands; 3 Department of Radiology, University Medical Center Utrecht, Utrecht, Netherlands; 4 Department of Radiology and Nuclear Medicine, Academic Medical Center, Amsterdam, Netherlands; 5 Centre for Big Data Research in Health, University of New South Wales, Sydney, NSW, Australia; 6 Department of Internal Medicine, Academic Medical Center, Amsterdam, Netherlands; 7 Department of Surgery, Academic Medical Center, Amsterdam, Netherlands; 8 Department Radiation Oncology, Academic Medical Center, Amsterdam, Netherlands; Bangladesh University of Engineering and Technology, BANGLADESH

## Abstract

The intravoxel incoherent motion (IVIM) model for diffusion-weighted imaging (DWI) MRI data bears much promise as a tool for visualizing tumours and monitoring treatment response. To improve the currently poor precision of IVIM, several fit algorithms have been suggested. In this work, we compared the performance of two Bayesian IVIM fit algorithms and four other IVIM fit algorithms for pancreatic cancer imaging. DWI data were acquired in 14 pancreatic cancer patients during two MRI examinations. Three different measures of performance of the fitting algorithms were assessed: (i) uniqueness of fit parameters (Spearman’s rho); (ii) precision (within-subject coefficient of variation, wCV); and (iii) contrast between tumour and normal-appearing pancreatic tissue. For the diffusivity D and perfusion fraction f, a Bayesian fit (IVIM-Bayesian-lin) offered the best trade-off between tumour contrast and precision. With the exception for IVIM-Bayesian-lin, all algorithms resulted in a very poor precision of the pseudo-diffusion coefficient D* with a wCV of more than 50%. The pseudo-diffusion coefficient D* of the Bayesian approaches were, however, significantly correlated with D and f. Therefore, the added value of fitting D* was considered limited in pancreatic cancer patients. The easier implemented least squares fit with fixed D* (IVIM-fixed) performed similar to IVIM-Bayesian-lin for f and D. In conclusion, the best performing IVIM fit algorithm was IVM-Bayesian-lin, but an easier to implement least squares fit with fixed D* performs similarly in pancreatic cancer patients.

## Introduction

The intravoxel incoherent motion (IVIM) model for diffusion-weighted imaging (DWI) data obtained by MRI bears much promise as a tool to both visualise and characterize tumours and to monitor treatment response (e.g. in radiotherapy or chemotherapy) [1–3]. Contrary to the classical DWI model, in which signal attenuation is modelled monoexponentially as a function of diffusion-weighting (b-value), the IVIM model predicts a biexponential decay, probing both tissue diffusion and perfusion. Since the introduction of the IVIM model [4], the non-monoexponential behaviour of DWI data in the pancreas was confirmed in multiple studies [1–3] and related to the interplay between diffusion and perfusion [5,6]. Consequently, the IVIM model has been used to delineate pancreatic cancer [3], characterise pancreatic lesions [1,2] and enabled treatment response monitoring in other organs [7,8].

One major challenge for IVIM is the limited precision of its parameters and relatively noisy perfusion maps [9–12]. To improve precision and obtain more homogenous maps, multiple algorithms for fitting IVIM model have been proposed. The performance of IVIM fit algorithms has been investigated in simulations [13–16] and volunteers[13,16] as well as several pathologies, such as brain[17], breast[18,19], rectum[20] and prostate[21] cancers.

For abdominal imaging, including pancreatic imaging, it was shown that the Bayesian fit, originally suggested by Neil and Bretthorst [22], gives the best results [23,24]. However, all pancreatic studies comparing fit algorithms were based either on simulations[16], healthy volunteer measurements [24] or in the healthy appearing liver tissue of patients with liver metastasis [23]. In data from pancreatic cancer patients, fitting may be more challenging due to the limited size of the tumour compared to the entire organ, lowered perfusion [1–3], and echo planar imaging (EPI) artifacts that occur as a result of e.g. air–tissue boundaries, intratumoral fiducials [25] or biliary stents [26]. Furthermore, the suggested Bayesian approaches are based on a data-driven prior. As the prior is joint over the separate model parameters, it can drive fits to certain combinations of fit parameter values, leading to strong artificial correlations between parameters. Furthermore, it can drive data to the more frequent occurring values in the prior (i.e. obtained in the larger healthy tissue), which has the potential to mask certain (smaller) pathologies, resulting in i.e. a decreased tumour contrast [15,16]. Therefore, it is important to assess the performance of such algorithms in cancer patients.

The objective of this exploratory study was to compare the performance of two Bayesian fitting algorithms with four other established IVIM fitting algorithms for pancreatic cancer imaging. We defined three criteria. First, for fitting parameters to render salient information, they should render unique information and, hence, have a limited correlation between each other. Second, in order to assess treatment response, the parameters from the fits should be precise [13,18,19]. Third, to delineate/detect tumours, a parameter with a high contrast between tumour and normal pancreatic tissue is desirable [27,28]. Prior-driven correlations between fit parameters should be picked-up by testing the uniqueness. If Bayesian algorithms’ prior drives the parameters (in the tumour) to mean values (from healthy tissue), it will have a decreased contrast. Finally, for Bayesian algorithms to have added value in monitoring treatment response they should show increased precision.

## Materials and methods

This prospective study (NCT01995240) was approved by the independent medical ethics committee of the Academic Medical Center Amsterdam (The Netherlands). All patients gave written informed consent. Inclusion criteria were: histopathological confirmed locally advanced or metastatic pancreatic ductal adenocarcinoma, normal kidney function (eGFR>60) and no contraindication to undergoing MRI. Sixteen consecutive patients fulfilling these criteria and willing to participate were included. Patients were scanned on a 3T scanner (Philips Ingenia, Best, The Netherlands; maximum gradient strength: 45 mT/m; maximum slew rate: 200T/m/s.) between October 2014 and March 2016 at our institute. Data were acquired with a 16-channel phased-array coil anterior 10-channel phased-array coil posterior to the patient. One patient dropped out between scan sessions, and for one patient, the scans were stopped due to patient discomfort. Thus, data from fourteen patients were analysed (eight females, mean age 67 years old, range 52–78, six males, mean age 70 years old, range 56–77). The same patient cohort was used to assess the precision of competing DWI models, using least squares fitting [29].

### Data acquisition

To enable assessment of inter-session and intra-session repeatability, all patients were scanned three times during two separate sessions (average: 4.5 days apart, range: 1–8 days). To minimise bowel motion hyoscine bromide (Buscopan, Boehringer, Ingelheim, Germany; 20 mg IV) was administered directly before the first DWI acquisition in each session. The data from the second acquisition within a session, for which no additional hyoscine bromide was administered right before scanning, were used for the intra-session analysis only.

For each patient, we acquired 2D multi-slice diffusion-weighted EPI data and contrast-enhanced (CE) T1-weighted multi-echo spoiled gradient echo (T1W GE) data with Dixon reconstruction (Table 1 shows imaging parameters). The T1W GE data were acquired 35s after Gadovist 1.0 (Bayer Healthcare, Leverkusen, Germany) administration (0.1ml/kg; 5ml/s, followed by 15 ml saline flush). DWI data were acquired in isotropic distributed directions per b-value. A small FOV was used to improve bandwidth of the sequence and minimise deformations of the anatomy. As the TE (and hence signal to noise ratio) of all b-value acquisitions is determined by the highest b-value acquisition, we chose to only acquire up to b = 600 s/mm^2^. This choice was based on previous work[9] and is justified by the short T2 of pancreatic tissue and the fact we are concentrating on the perfusion related effects on the signal present in signal from b<150 s/mm^2^.

**Table 1:** Sequence parameters.

	DWI	T1W GE
**FOV (RL × AP) (mm**^**2**^**)**	432 × 108	400 × 353
**Acquisition matrix**	144 × 34	236 × 208
**Slices**	18	56
**Slice thickness/gap (mm)**	3.7/0.3	1.7/-
**TR**^1^**/TE/ ΔTE (ms)**	>2200/45/-	4.7/1.15/1.0
**FA (°)**	90	10
**BW (Hz/voxel)**	59 (phase direction)	1602 (frequency)
**Parallel imaging**	1.3 (AP)	2/1.5 (RL/AP)
**Partial Fourier**	0.8	no
**Respiratory compensation**	Respiratory trigger (navigator)	1 breath hold
**Fat saturation**	Gradient reversal during slice selection + SPIR	Dixon reconstruction
**b-values (s/mm**^**2**^**) and directions (between brackets)**^2^	0 (15), 10 (9), 20 (9), 30 (9), 40 (9), 50 (9), 75 (4), 100 (12), 150 (4), 250 (4), 400 (4) and 600 (16)	
**Diffusion times δ/Δ (ms)**	10.1/22.6	

^1^TR of the DWI acquisition was determined by the respiratory trigger interval, but it was at least 2200 ms.

^2^ numbers between brackets indicate number of directions.

DWI, diffusion-weighted imaging; FOV, Field of view; RL, right-left; AP, anterior-posterior; ΔTE, increase in TE; FA, flip angle; BW, bandwidth per voxel; SPIR, spectral presaturation with inversion recovery; δ, diffusion gradient time; Δ delay time between diffusion gradient onsets.

### Post processing

All data analysis, fitting and statistical tests were performed in Matlab 2013a (MathWorks, Natick, U.S.A.), except for the IVIM-Bayesian-log algorithm, which was implemented in DTITools for Mathematica [30], Mathematica 10.4.1 (Wolfram Research, Champaign, U.S.A.).

All DWI images were denoised using a Rician adaptive non-local means filter [31], developed for spatially varying noise due to i.e. parallel imaging, and registered in Elastix [32,33] S1 File for details).

We tested two Bayesian algorithms: IVIM-Bayesian-log [23,34] and IVIM-Bayesian-lin [24]. The IVIM-Bayesian-log was an implementation from the Bayesian approach described in detail by Orton et al.[23], which we implemented in Mathematica (‘BayesianIVIMFit2’ from DTITools [30], available at https://github.com/mfroeling/DTITools). The IVIM-Bayesian-lin used the Matlab scripts from Barbieri et al. [24]. Both algorithms are described in detail in the referenced articles. For IVIM-Bayesian-log the prior was defined over the transformed parameters f = log(F)-log(1-F), d = log(D) and d* = log(D*), in order to constrain the parameters to a physical domain. The prior was then set as a multivariate Gaussian distribution over these parameters. For IVIM-Bayesian-lin, no log transformation was taken and the joint prior probability was set to a uniform distribution over the restricted parameter space while no Gaussianity was assumed.

The Bayesian approaches were compared with four alternative fitting algorithms: IVIM-free, IVIM-adaptive [35], IVIM-MLE [36] and IVIM-fixed (Table 2 for details). The IVIM-free and IVIM-fixed fitting algorithms were implemented in Matlab using the ‘fit’ function from the curve fitting toolbox. The ‘NonlinearLeastSquares’ method was used to apply a voxel-wise non-linear least squares fit of the IVIM model to the DWI data. The IVIM-MLE used the ‘fit_mri’ function from the ‘fit MRI package’ toolbox from Poot et al. in Matlab [36] to apply a voxel-wise maximum likelihood estimator based fit of the IVIM model to the DWI data.

**Table 2:** Fit algorithms.

Name	Fit
**IVIM-Bayesian-log** [23,34]	Data-driven Bayesian algorithm for which the prior is a fitted Gaussian in log-space to confine parameters to relevant values
**IVIM-Bayesian-lin [24]**	Data-driven Bayesian algorithm using boxcar functions with support over pre-defined ranges as weakly informative priors.
**IVIM-free**	Levenberg-Marquardt algorithm for a least squares fit
**IVIM-adaptive** [35]	Adaptive threshold segmented fit
**IVIM-MLE** [36]	Maximum likelihood estimator algorithm which assumed Rician noise
**IVIM-fixed**	Levenberg-Marquardt algorithm for a least squares fit, except that D* was fixed to 70×10^−3^ mm^2^/s, which resulted in more stable fits in healthy volunteers [9] (value based on volunteer data).

All IVIM model fit algorithms converted the IVIM signal fractions into blood volume fractions using the formula by Lemke et al. [6] (formula 2 in reference). This conversion required using our TE = 45 ms and assuming a TR = 5000 ms (typical respiratory cycle), T_1_ = 725 ms and T_2_ = 43 ms for the pancreas and T_1_ = 1932 ms and T_2_ = 275 ms for blood [37,38]. To improve precision [13], fit parameters were constrained in all fits as follow: 0.5×10^−3^<D<6×10^−3^ mm^2^/s, 6×10^−3^ <D*<200×10^−3^ mm^2^/s, 0.1<f<99%. D had no constraints in the IVIM-adaptive approach; IVIM-Bayesian-log had the following constraints: D>0 mm^2^/s, D*>0 mm^2^/s and 0%<f<100%.

**Fig 1:**
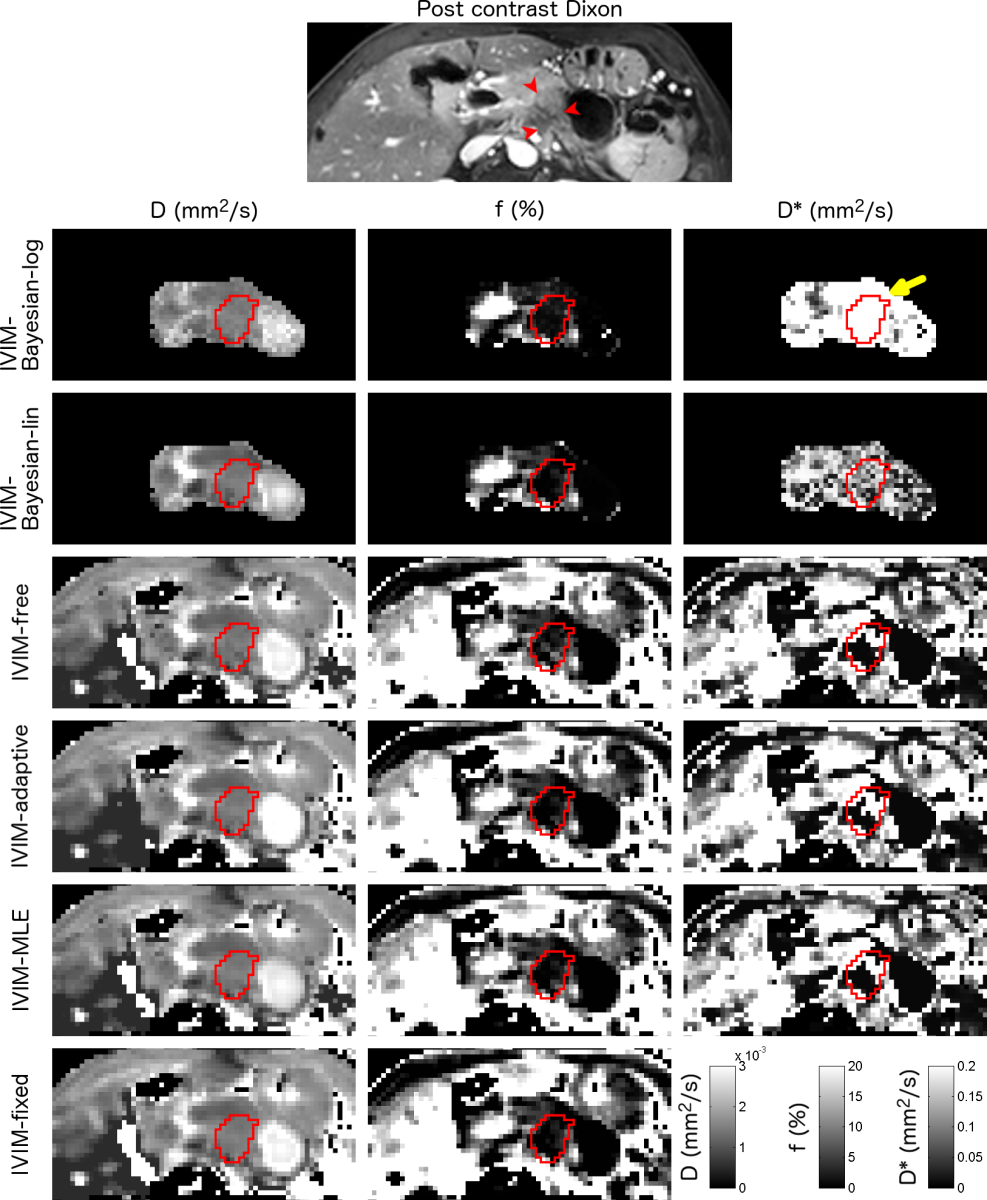
Example parameter maps from a pancreatic cancer patient. Axial parameter maps of the fit parameters of six IVIM model fit algorithms in a 60-year-old female with pancreatic adenocarcinoma in the pancreas tail. ROIs containing pancreatic tumour are shown. The CE T1W GE is added as a reference. Note that for the Bayesian approaches, not all voxels were fitted, as including more voxels will influence the prior. This patient had D, f and D* between 1.1–1.5×10^−3^ mm^2^/s, 1.1–2.1% and 116–989×10^−3^ mm^2^/s respectively. The yellow highlights the high D* values fitted in IVIM-Bayesian-log, compared to the other fit algorithms.

**Fig 2:**
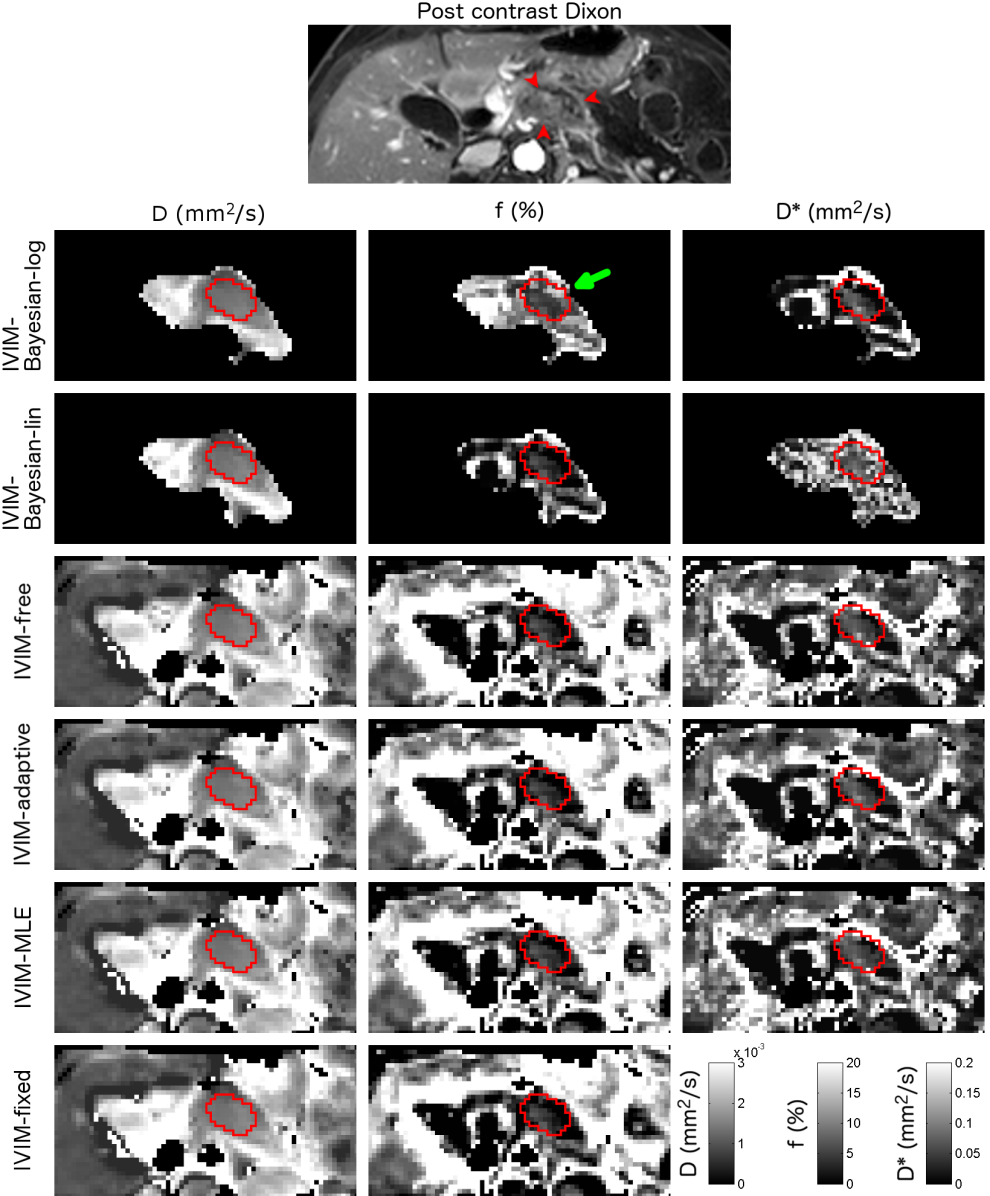
Example parameter maps from a pancreatic cancer patient. Axial parameter maps of the fit parameters of six IVIM model fit algorithms in a 61-year-old female with pancreatic adenocarcinoma in the pancreas corpus. ROIs containing pancreatic tumour are shown. The CE T1W GE is added as a reference. Note that for the Bayesian approaches, not all voxels were fitted, as including more voxels will influence the prior. This patient had D, f and D* between 1.3–1.5×10^−3^ mm^2^/s, 2.1–6.6% and 43–98×10^−3^ mm^2^/s respectively. The green arrow highlights the higher f found in IVIM-Bayesian compared to the other algorithms.

**Fig 3:**
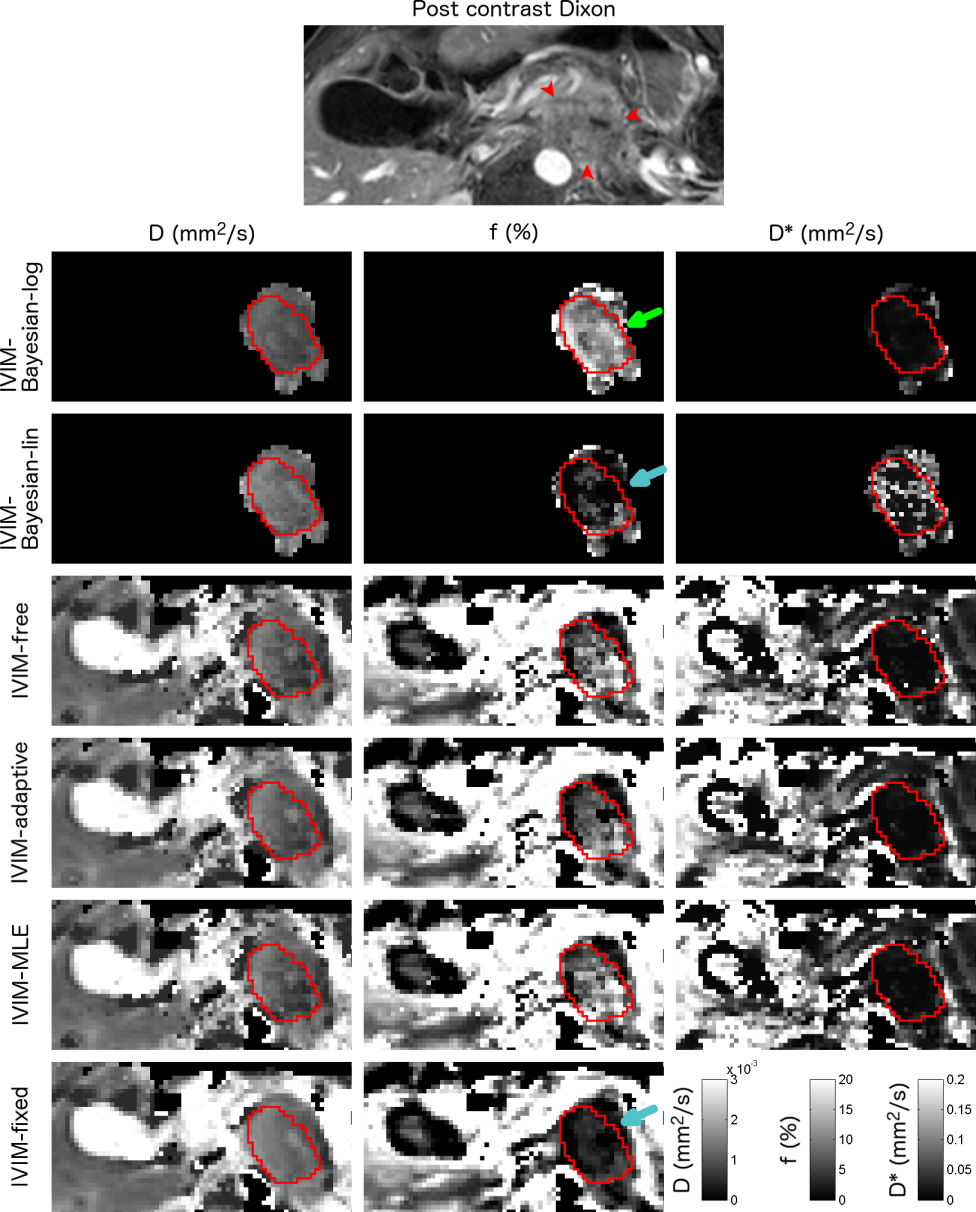
Example parameter maps from a pancreatic cancer patient. Axial parameter maps of the fit parameters of six IVIM model fit algorithms in a 71-year-old male with pancreatic adenocarcinoma in the pancreas tail. ROIs containing pancreatic tumour are shown. The CE T1W GE is added as a reference. Note that for the Bayesian approaches, not all voxels were fitted, as including more voxels will influence the prior. This patient had D, f and D* between 1.0–1.3×10^−3^ mm^2^/s, 2.0–14.9% and 15–70×10^−3^ mm^2^/s respectively. The green arrow highlights the higher f found in IVIM-Bayesian compared to the other algorithms. The blue arrows highlight the lower f in IVIM-Bayesian-lin and IVIM-fix.

An abdominal radiologist (M.R.W.E., 9 years’ experience) and an abdominal imaging researcher (R.K. 3 years’ experience) drew regions of interest (ROIs) in consensus using 3D Slicer [39]. ROIs had a minimum size of 100 voxels and comprised a minimum of three slices. For each patient, two ROIs were created per scan, one containing pancreatic tumour tissue and one containing normal-appearing pancreatic tissue. The ROIs were drawn on an ADC-map, generated from b = 0 s/mm^2^ and 600 s/mm^2^, under the guidance of CE T1W GE images. ROIs were drawn freely and care was taken to include as much tumour or normal pancreatic tissue in the ROI as could be reliably determined based on imaging characteristics. The mean value of the voxel-wise fits within the ROIs was calculated. Figs 1–3 shows an example of an ROI.

In the data-driven Bayesian algorithms used, fits were done per patient and their priors were defined using all voxels within the ROI used for fitting. If the entire FOV would be included, the prior would consist of a lot of different tissue types and the prior might be contaminated with other types of tissue. Therefore a new ROI was defined within which the Bayesian algorithms were plotted. To incorporate sufficient voxels within this ROI to estimate the prior, a researcher (O.J.G) delineated a very rough outline of the entire pancreas and tumour, including parts of the neighbouring organs, to be fitted by the Bayesian algorithms. After fitting to data within the rough ROI, the abovementioned well-defined ROIs, delineated in consensus by the experienced radiologist, were applied.

### Comparison of methods

To spot any shortcoming the Bayesian approaches might display, we quantitatively evaluated the performance of the six IVIM model fitting algorithms for pancreatic cancer imaging considering the following three factors. To identify any prior-driven correlations between fit parameters we assess parameter uniqueness. To evaluate whether Bayesian algorithms could have added value in monitoring treatment outcome, it’s precision is tested. Finally, to test whether Bayesian algorithms prior drives parameters to a mean value, the contrast between tumour and normal-appearing pancreatic tissue is assessed.

#### Uniqueness

We used a Spearman’s rank correlation test between the fit parameters to examine the unique nature of the fit parameters (significance level α = 0.05). For this purpose, only fit parameters from the first acquisition per patient are considered. Fit parameter combinations with significant Spearman’s rho indicate both parameter values are significantly correlated and hence determining both parameters has limited added value.

#### Precision

From the repeated measures we calculated the inter- and intra-session within-subject coefficient of variation (wCV) of the tumour ROI as a measure of precision [40]. A low wCV indicates stable parameter values without intervention, which is desirable for treatment response monitoring. Per parameter, Wilcoxon signed-rank tests tested whether the remaining parameters had significantly higher wCV than the parameter with lowest wCV. The Wilcoxon signed-rank test was performed over the squared differences of the repeated measure (m_1_ and m_2_), divided by the squared mean (μ) of the population for that parameter: (m_1_-m_2_)^2^/μ^2^ (significance level α = 0.05).

#### Tumour contrast

As noise is poorly defined in an IVIM model parameter map, it was challenging to obtain contrast to noise ratio. Therefore, contrast was calculated as the difference in parameter value between the tumour and normal-appearing pancreas. To normalize the contrast to some reference, such that contrast in D (in the order of 10^−4^) can be compared with contrast in f (in the order of 0.1), the difference is divided by the mean parameter value and multiplied by 100%. Hence, contrast was defined as the percentage difference in parameter value between tumour and normal-appearing pancreas tissue. A higher tumour contrast indicates a parameter enables for better distinguishing between tumour and normal-appearing pancreatic tissue. To test whether parameters had significantly lower contrast than the parameter with the highest contrast, a Wilcoxon signed-rank test was performed over the contrasts per patient (significance level α = 0.05).

Finally, we plotted precision (inter-session wCV) as a function of contrast.

## Results

In two out of forty-two acquisitions it was not possible to delineate the tumour in the repeated intra-session scan. Therefore, intra-session wCVs were determined using twelve patients. In one patient, no normal-appearing pancreatic tissue was present in all repeated images and in two patients no normal-appearing pancreatic tissue was present in one of the scan sessions. Therefore, tumour contrast was based on thirteen patients (of which 2 had only healthy tissue in 1 scan session). The mean mask sizes were 7.6 cm^3^ = 210 voxels, range 3.0–23.5 cm^3^) for the tumour ROIs and 4.2 cm^3^ = 118 voxels, range 1.3–8.1 cm^3^) for the normal-appearing pancreatic tissue ROIs.

Parameter maps were generated for all fit algorithms (Figs 1–3). Table A in S3 File shows the average fitted parameter values. The IVIM-Bayesian-log algorithm gave different parameter values than the other algorithms, in particular for f and D*. For example, f from IVIM-Bayesian-log was 7.56%, whereas it was 2.56–4.98% for all other algorithms in tumour tissue (highlighted i.e. by the green arrow in Figs 2 and 3) and D* was 208×10^−3^ mm^2^/s (i.e. highlighted by the yellow arrow in Fig 1), compared to 61.1–83.5×10^−3^ mm^2^/s for all other algorithms. Furthermore, f was often lower in IVIM-fixed and IVIM-Bayesian-lin than in the other algorithms (i.e. blue arrows in Fig 3).

### Uniqueness

**Fig 4:**
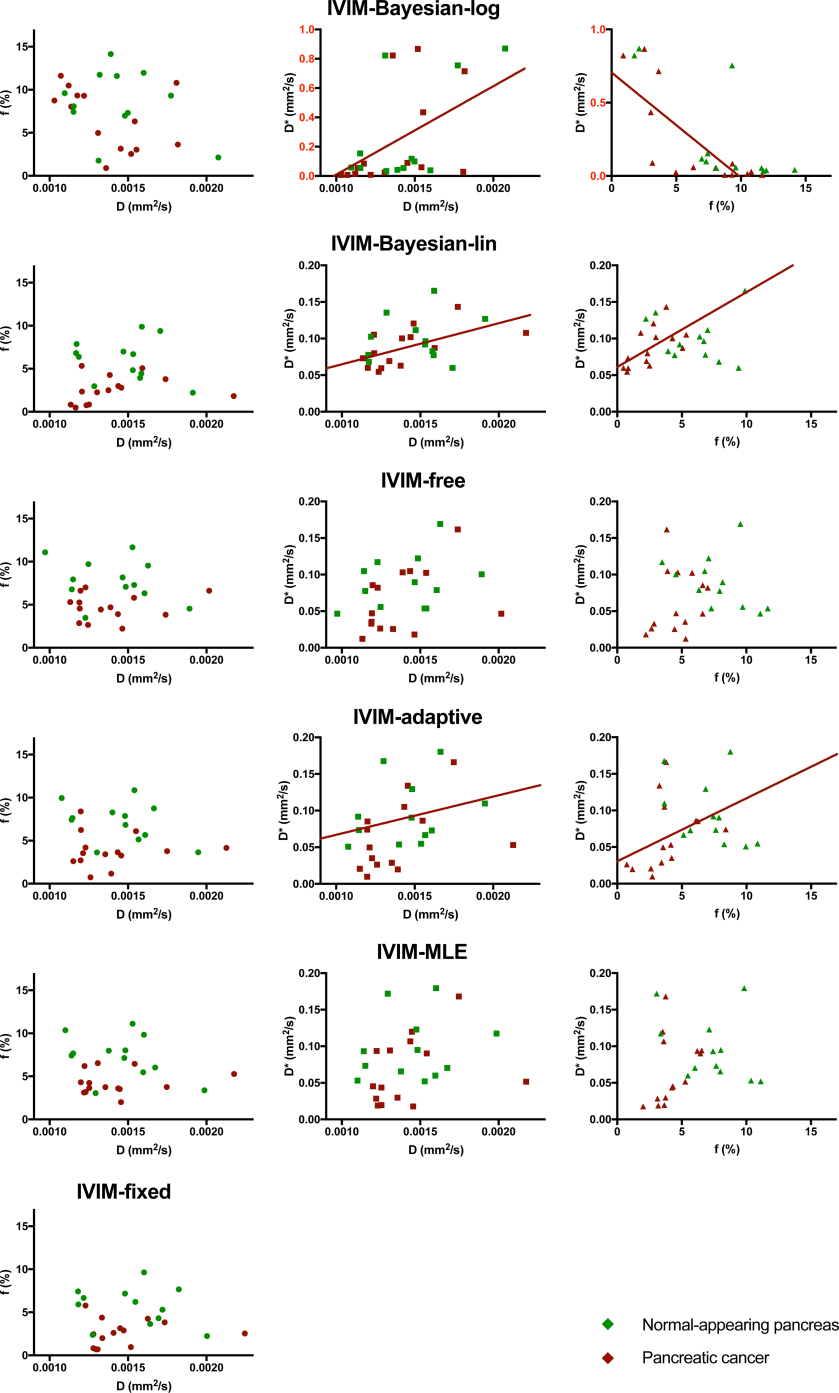
Correlations between fit parameters. Correlation between fit parameters D&f (left column), D&D* (middle column) and f&D* (right column). For the parameter pairs with significant correlation according to Spearman’s rank correlation, a linear regression line is plotted to the data from pancreatic cancer. The correlations were only tested using pancreatic cancer tissue (dark red dots). Note that the D*-axis of IVIM-Bayesian-log is stretched to fit all data and hence highlighted in red.

There was a significant correlation for D&D* and f&D* of IVIM-Bayesian-log, IVIM-Bayesian-lin and IVIM-adaptive, indicating that the D* parameter is of limited added value in these algorithms (Table 3, Fig 4). There were no significant D&D* and f&D* for the IVIM-free and IVIM-MLE approach. D&f showed no significant correlation for any of the fit algorithms. Despite being non-significant for D&f (p = 0.084–0.104), Spearman’s rho was up to a factor five higher for D&f from the Bayesian algorithms (absolute values 0.45–0.48) compared to D&f from the non-Bayesian algorithms (absolute values 0.09–0.22).

**Table 3:** Uniqueness.

	Spearman’s rho			p-value		
	D&f	D&D*	f&D*	D&f	D&D*	f&D*
**IVIM-Bayesian-log**	-0.48	0.68 *	-0.81 *	0.084	0.009	<0.001
**IVIM-Bayesian-lin**	0.45	0.61 *	0.67 *	0.104	0.022	0.011
**IVIM-free**	-0.09	0.45	0.24	0.762	0.112	0.417
**IVIM-adaptive**	0.12	0.57 *	0.59 *	0.682	0.035	0.030
**IVIM-MLE**	0.10	0.37	0.47	0.739	0.192	0.904
**IVIM-fixed**	0.22			0.454		

* = significant correlation (p<0.05)

### Precision

The inter-session wCVs (Table 4) were on average 30% larger than the intra-session wCVs (Table B in S3 File), indicating a larger test-retest variation when scans are repeated on separate days compared to repeated in the same scan session. IVIM-Bayesian-lin had most repeatable f and D*, and the wCV for D was not significantly higher (worse) than the best wCV for D (IVIM-fixed; Table 4). IVIM-fixed had most repeatable D, and its f did not have significantly worse repeatability than IVIM-Bayesian-lin.

**Table 4:** Precision of parameters.

	Inter-session wCV			p-value		
**Fit algorithms**	D	f	D*	D	f	D*
**IVIM-Bayesian-log**	12.6	52.2 *	159.4 *	0.104	**0.035**	**0.035**
**IVIM-Bayesian-lin**	7.2	**25.7**	**24.2**	0.153	Best	Best
**IVIM-free**	10.0 *	40.9	50.5 **	**0.030**	0.463	**0.007**
**IVIM-adaptive**	8.5 *	34.4	51.9 *	**0.020**	0.104	**0.017**
**IVIM-MLE**	8.4 *	35.8	52.7 *	**0.042**	0.268	**0.011**
**IVIM-fixed**	**6.7**	28.7		Best	0.761	

wCV, within-subject coefficient of variation.

In the left four columns show the wCV of the different parameters. The parameter with lowest wCV is printed bold. Stars indicate the parameters that were significantly

(*: p<0.05, **: p<0.01)

worse (Wilcoxon signed-rank) than the best scoring parameter of the three groups (D, f, and D*).

The right three columns list the p-values of the Wilcoxon signed-rank test between the algorithm with lowest wCV (Best) of each parameter and the other algorithms. Bold values indicate p-values belonging to significantly worse values.

### Tumour contrast

IVIM-Bayesian-lin showed the highest contrast for both D and f, with f having significantly more contrast (contrast = 93.7%) than f from all other algorithms (Table 5). For all algorithms the contrast in f was much (7–96 times) larger than the contrast in D. The contrast in D* was highest in IVIM-adaptive but of the same order of magnitude as the inter-session wCV of D*.

**Table 5:** Contrast.

	Contrast (%)			p-value		
**Fit algorithms**	D	f	D*	D	f	D*
**IVIM-Bayesian-log**	4.3	30.8 **	36.4	0.080	**<0.001**	0.414
**IVIM-Bayesian-lin**	**4.7**	**93.7**	17.2 **	Best	Best	**<0.001**
**IVIM-free**	0.7	56.7 **	50.5	0.305	**<0.001**	0.787
**IVIM-adaptive**	2.7	70.8 **	**52.9**	0.685	**<0.001**	Best
**IVIM-MLE**	4.3	61.0 **	47.8	0.216	**<0.001**	0.191
**IVIM-fixed**	0.9	87.8 **		0.787	**0.003**	

In the left four columns show the contrast between the pancreatic tumour and the normal-appearing tissue for the different parameters. The parameter with lowest contrast is printed bold. Stars indicate the parameters that were significantly

(**: p<0.01)

worse (Wilcoxon signed-rank) than the best scoring parameter of the three groups

(D, f, and D*).

The right three columns list the p-values of the Wilcoxon signed-rank test between the algorithm with lowest wCV (Best) of each parameter and the other algorithms. Bold values indicate p-values belonging to significantly worse values.

**Fig 5:**
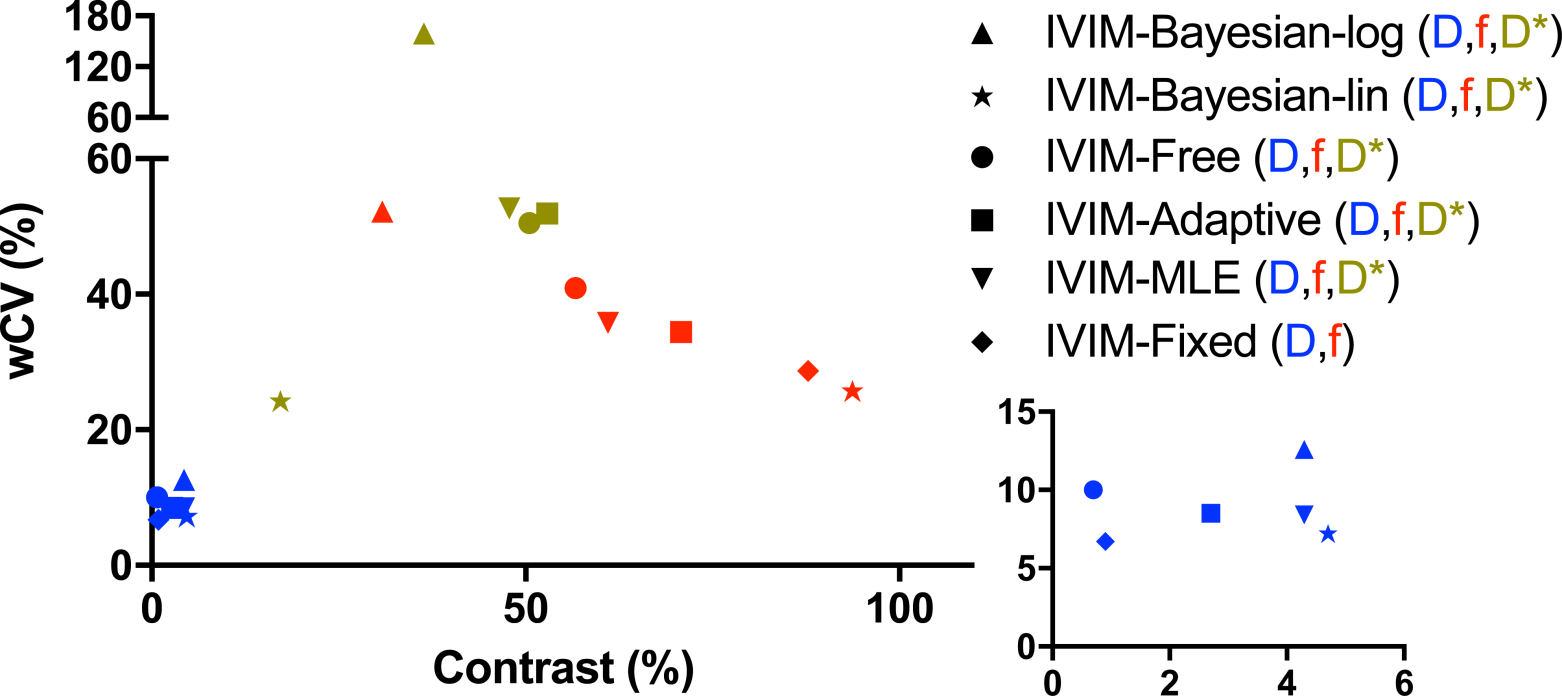
wCV vs contrast. Plots of the inter-session wCV as a function of contrast between tumour and pancreatic tissue for the diffusion-related parameter (blue) and other fit parameters (red, green). Bottom right graph has zoomed to low wCV and contrast to illustrate the trade-off for D.

Ideally, a parameter has a high contrast and a low wCV. When wCV is plotted as function of contrast (Fig 5), it is apparent that f from IVIM-Bayesian-lin has the overall best relation between contrast and wCV, followed closely by f from IVIM-fix. When D is of interest, again IVIM-Bayesian-lin shows the overall best trade-off between wCV and contrast. D* shows a poor trade-off between wCV and contrast. It is interesting to note that IVIM-Bayesian-lin has a relatively low wCV and contrast in D* compared to the other algorithms.

## Discussion

We evaluated the performance of two Bayesian fit algorithms for the IVIM model and compared them with four other fit algorithms in patients with pancreatic cancer. We established the uniqueness and precision of the fit parameters, and the contrast between tumour and normal-appearing pancreatic tissue. Considering both D and f, the IVIM-Bayesian-lin performed best as it exhibited the highest contrast for both parameters and had the highest precision for f while having similar precision for D compared to the other algorithms. IVIM-Bayesian-lin showed a significant correlation between D* and the other fit parameters. Yet, for all other algorithms, the added value of pseudo-diffusion coefficient D* was also limited, as the precision was low (IVIM-Bayesian-log, IVIM-free, IVIM-adaptive and IVIM-MLE) and/or the parameter was correlated to f and D (IVIM-Bayesian-log, IVIM-Bayesian-lin and IVIM-adaptive). The other Bayesian algorithm (IVIM-Bayesian-log) performed worst of all considering uniqueness (D&f, D&D*, f&D*), precision (D, f, D*) and contrast (f). This stresses the importance of testing fit algorithms in patient data before implementing them clinically. The best algorithm, IVIM-Bayesian-lin, did not significantly outperform IVIM-fixed, the second best algorithm, considering both precision and contrast of f and D. So, all in all, IVIM-Bayesian-lin is preferred, however, IVIM-fixed might be a strong, easier to implement, alternative to the Bayesian algorithm.

For all IVIM fit algorithms, the tumour contrast was more prominent in the perfusion fraction f, whereas diffusivity D was more precise. Whether D* has an added value for IVIM of pancreatic cancer patients is debatable. For all non-Bayesian algorithms the precision of D* is very poor and D* images look very noisy (Table 4, Figs 1–3). For all Bayesian algorithms, D* had strong correlations with the other fit parameters (Table 3, Fig 4). This implies a limited added value of D*. However, from Fig 4 suggests that to distinguish between tumour and healthy tissue, the combination of D* and f might help, where high D*+f relate to normal-appearing tissue and low D*+f relate to tumours.

From the fit algorithms tested, IVIM-Bayesian-lin performed best considering precision and tumour contrast (Tables 4 and 5, Fig 5). IVIM-Bayesian-lin showed the highest precision for f and D* compared to the other algorithms, and the precision of D was not significantly lower compared to the algorithm with the highest precision of D (IVIM-fixed). Furthermore, the contrast between tumour and normal-appearing pancreatic tissue generated by f was significantly higher than in all other algorithms. These findings are in agreement with earlier published simulations and volunteer measurements [24].

However, IVIM-Bayesian-lin is not widely implemented. Therefore, IVIM-fixed may be a good alternative (Tables 4 and 5, Fig 5). IVIM-fixed showed the highest precision of D, and the precision in f was second best and not significantly worse than IVIM-Bayesian-lin. Also, the contrast in f was second best, and though significant, it was only 7% lower than IVIM-Bayesian-lin. A disadvantage of IVIM-fixed is that no information on D* is obtained.

IVIM-Bayesian-log scored worst of all algorithms. We believe there are two reasons for the discrepancy between the performances of both Bayesian approaches. First, contrary to IVIM-Bayesian-lin, IVIM-Bayesian-log fitted a Gaussian distribution to the data-driven prior which could centre the prior on parameter values typical for other tissues contained in the ROI (i.e. around normal-appearing pancreatic values instead of tumorous values). Secondly, the log transforms in IVIM-Bayesian-log decrease the probability of low parameter values as they are spread out in log space and, ultimately, a value of 0 will be impossible as it translates to infinity in log-space. As pancreatic cancer is poorly perfused, this could have played a large role in these patients. This is reflected in the relative high perfusion fraction found for IVIM-Bayesian-log compared to the other fit algorithms and can be seen in Figs 2 and 3 (green arrow). In previous studies[15,23] IVIM-Bayesian-log performed better than in our study. Compared with these studies, our in vivo perfusion fractions were low. In particular, the study by Orton et al. [23] showed the performance of IVIM-Bayesian-log only in well perfused liver. Potentially, IVIM-Bayesian-log can perform better in well-perfused tumours.

When one desires to perform treatment evaluation and response monitoring, two aspects must be taken into account. First, the relevant model parameter needs to be measured with high precision and hence a low wCV. Second, there should be a change in the parameter of interest as a result of the treatment. Tissue diffusion has been reported as a good biomarker for treatment response for e.g. responders to chemotherapy of colorectal hepatic metastasis had increased diffusivity (1.41×10^−3^ mm^2^/s vs 1.15×10^−3^ mm^2^/s) [41]. The parameter with lowest wCV was D of the IVIM-fixed fit approach (Table 4). The inter-session wCV of D from IVIM-Bayesian-lin was similar (i.e. non-significant, 13% worse). Perfusion-related parameters may be more sensitive to probe angiogenic changes as a result of therapy [7,8]. In such a situation, f or D* might probe changes. As D* was poorly repeatable or not unique in the tested algorithms, f is considered here. IVIM-Bayesian-lin had the best precision in f (inter-session wCV = 26%); however, IVIM-fixed had a very similar value (inter-session wCV = 26%). Both are relatively high. Hence, when monitoring individual treatment response, one should be aware of the limited precision of the perfusion-related parameters.

Bayesian algorithm for fitting may introduce a bias to the results [15,42]. From simulations it was shown that the bias in IVIM-Bayesian-lin [24] as well as IVIM-Bayesian-log [16] was low. In our results, we find that IVIM-Bayesian-lin gives parameter values in a similar range of the parameter values from the non-Bayesian algorithms (Table A in S3 File) both in tumorous tissue and normal-appearing pancreatic tissue. However, the IVIM-Bayesian-log gives very different parameter values than the other tested algorithms (Table A in S3 File, green and yellow arrows in Figs 1–3). Therefore, we believe that IVIM-Bayesian-log can cause bias in the results for pancreatic cancer patients. This is most likely a result of the Gaussian prior from the log-transformed parameters, which made it hard to result in very low f. As a result, where f, in reality, might be zero, the Bayesian fit will force it to non-zero values. To account for the fit being forced bi-exponentially (non-zero f) while data might be described better mono-exponentially, D* is increased by the fit algorithm. This is illustrated in Fig 4 (top right graph), where low f data points have very high D* values, whereas patients with higher f values have D* values similar to other algorithms.

It was previously shown that fit constraints can improve the precision of fits [13]. To have a fair comparison between fitting algorithms, we kept the fit constraints similar for most algorithms. In IVIM-adaptive, D is determined only by a mono-exponential fit to high b-values and hence more stable; therefore it was not constrained. In IVIM-Bayesian-log the constraints are imposed by single, or double logarithmic transformations of the fit parameters, limiting the constraint range. Our constraints were chosen heuristically. D was not strongly constrained as this parameter has good precision. The constraints of f were 0.1–99%, as these values naturally occurred in the tissue. We constrained D* more sternly (6×10^−3^–200×10^−3^ mm^2^/s). With the chosen lowest non-zero b-value of 10 s/mm^2^, we are not able to accurately distinguish D*>200×10^−3^ mm^2^/s. The lower end of D* coincided with the maximum for D. Potentially, parameters could have been constrained more than in this work, to better guide the fits and find more reproducible results. For example, there is a continuous set of options between fully fixing D* (IVIM-fix) and constraining it to a narrower set within IVIM-free. Finding the ideal constraints falls outside the scope of this research.

We believe that the good performance of IVIM-Bayesian-lin can be generalized to other tumour sites. For the IVIM-Bayesian-lin approach, it was illustrated that it outperformed several fit algorithms in healthy abdominal organs [24]. As the Bayesian approaches used data-driven priors, the homogeneity of healthy organs can be favourable for these algorithms. Although the heterogeneity of tumours could potentially decrease the performance, we did not encounter such issues in this study. We believe this illustrates that it would be the preferred algorithm for imaging of most tumour sites. A note needs to be added that IVIM-Bayesian-lin might fail for very small tumours, as the contribution of the tumour to the prior might be limited. In this case, the prior could be defined from data from multiple patients. For larger organs, such as the liver, including the entire organ and its surroundings to the prior (as done in pancreas patients, Figs 1–3) might cause the prior to be overly determined by non-tumorous tissue, which could also influence the performance.

Note that there was a large spread in parameter values among all algorithms, including among the non-Bayesian algorithms, in particular for f (overall mean f: 2.60–4.98%). Specially, in Fig 3 one can see a patient for which IVIM-Bayesian-log gives a very large f (14.9%; green arrow) whereas the IVIM-fixed and IVIM-Bayesian-Lin give a low f (2.0–2.2%, blue arrows). The other algorithms had intermediate f (4.4%–5.1%). This should be considered when comparing results from different studies using different fit algorithms.

### Limitations

A limitation of this study was that the ROI delineations were based on CE T1W GE and ADC maps from b = 0 and 600 s/mm^2^. Therefore, the tumorous ROI contained regions with low perfusion (CE T1W GE, ADC-map) and, potentially, diffusion restriction (ADC-map). However, so far, this is considered the best way to delineate pancreatic tumours.

In this study, we chose to assess six fit algorithms. However, this list is not conclusive and there are multiple other fit algorithms available. We believe the main streams of fit algorithms are discussed in this work and we do not think other algorithms will greatly improve the results compared to the ones presented in this work.

Furthermore, there are multiple competing models that might better describe DWI data in the tumour than IVIM. It was shown that two mono-exponential fits worked equally well as a bi-exponential fit for detecting treatment associated changes in parameter values after radiotherapy of pancreatic cancer patients [29]. This was partially attributed to the fact that the bi-exponential fit had poorer precision. However, this research was conducted using least squares fitting only and using Bayesian approach might improve the precision enough to detect more significant changes.

Finally, we have only assessed the parameters in tumorous tissue and normal-appearing pancreatic tissue. Possibly other tissues should be considered too when deciding which algorithm to use, such as cystic and necrotic regions, and pancreatic or bile ducts.

### Conclusions

The data-driven Bayesian algorithm IVIM-Bayesian-lin gives the best results for IVIM modelling of pancreatic cancer DWI data. This fit approach performed best considering the precision and contrast of most of the fit parameters. However, the added value of the D* estimate is limited as it is strongly correlated to the values of D and f. Therefore, the easier implemented least squares fit where D* is set to a fixed value prior to fitting the IVIM model to the DWI data is a strong alternative as it had similar precision and contrast. The other tested Bayesian fit algorithm, IVIM-Bayesian-log, performed worst of all tested algorithms. This result stresses the importance of testing a Bayesian algorithm on the desired pathology before implementing it clinically.

## Supporting information

S1 File Image protocol and post-processing details. (PDF)

S2 File IVIM algorithm fitting details. (PDF)

S3 File Two tables showing mean parameter values and intra-session wCV. (PDF)

S1 Data The raw data from the tumour and normal-appearing tissue ROIs (signal intensity per b-value). (ZIP)
