# Supplementary material for: Comparison of six fit algorithms for the intra-voxel incoherent motion model of diffusion-weighted magnetic resonance imaging data of pancreatic cancer patients
Source: PLoS One. 2018 Apr 4;13(4):e0194590. doi: 10.1371/journal.pone.0194590 (PMC5884505; doi:10.1371/journal.pone.0194590)
Supplement: S1 File — (PDF) [file pone.0194590.s001.pdf]

# Supporting information I

DWI was acquired using respiratory triggering, acquiring one b-value in one diffusion direction per trigger. All slices of the volume were acquired during one trigger to avoid inter-trigger mismatches between slices of one single volume. This approach resulted in long acquisition periods each trigger (1.8 s). Patients were instructed to hold their breath during the typical noise produced by the EPI readout and to breathe freely during the navigator acquisition to minimise intra-trigger respiratory motion.

All DWI images were denoised using a Rician adaptive non-local means filter [31], with a search radius of three voxels and a patch radius of one voxel. The images were registered using a two-step approach in Elastix [32]. First, a reference image was created by averaging at least five manually selected acquisitions from the same respiratory position. To correct for bulk displacements between acquisitions, we performed a mutual information based rigid Euler transformation on each b-value and direction to this reference image. The second step used a non-rigid b-spline registration based on mutual information to adjust for further deformations. During this step, we used two registration approaches: a single group-wise 4D [33] registration, or multiple 3D registrations for each b-value. We manually selected the registration approach that resulted in the most stable anatomy across the images acquired at different b-values and gradient directions (assessed visually, qualitatively).

## *References:*

31. Manjón J V, Coupé P, Martí-Bonmatí L, Collins DL, Robles M. Adaptive non-local means denoising of MR images with spatially varying noise levels. J Magn Reson Imaging. 2010;31: 192–203. doi:10.1002/jmri.22003

32. Klein S, Staring M, Murphy K, Viergever MA, Pluim JPW. Elastix: A toolbox for intensity-based medical image registration. IEEE Trans Med Imaging. 2010;29: 196–205. doi:10.1109/TMI.2009.2035616

33. Huizinga W, Poot DHJ, Guyader JM, Klaassen R, Coolen BF, Van Kranenburg M, et al. PCA-based groupwise image registration for quantitative MRI. Med Image Anal. Elsevier B.V.; 2016;29: 65–78. doi:10.1016/j.media.2015.12.004
