# Supplementary material for: Comparison of six fit algorithms for the intra-voxel incoherent motion model of diffusion-weighted magnetic resonance imaging data of pancreatic cancer patients
Source: PLoS One. 2018 Apr 4;13(4):e0194590. doi: 10.1371/journal.pone.0194590 (PMC5884505; doi:10.1371/journal.pone.0194590)
Supplement: S2 File — (PDF) [file pone.0194590.s002.pdf]

Supporting information II

Details on the IVIM fit algorithms are described in Table 2. Note that S_0 was fitted too, instead of fixed to value from the b=0 s/mm^2 acquisition. However, results of S_0 were not analysed.

Per b-value, we averaged the data to increase fitting speed. The mean data from b-values obtained in 4, 9, 12, 15 and 16 evenly distributed directions were weighted 1, 2, 3, 4 and 4 times during the fit, respectively. All fits were done voxel-wise. The directional analysis falls outside the scope of this research.

Voxels with a large value of the IVIM model parameter f contained mainly large vessels with instantaneous signal decay [9]. These voxels which greatly influences f and D* in the IVIM model. As the signal decay in these voxels are not due to capillary perfusion, which is what f and D* are supposed to describe, we removed voxels with f>25% when determining mean f and D* from the IVIM algorithms. In addition, when f<1%, there is too little perfusion signal to determine D*. Therefore, only signal from voxels with f>1% are considered when calculating mean D* from ROIs. The selection of voxels excluded due to these criteria was done for each algorithm individually.

References

Gurney-Champion OJ, Froeling M, Klaassen R, Runge JH, Bel A, Van Laarhoven HWM, et al. Minimizing the Acquisition Time for Intravoxel Incoherent Motion Magnetic Resonance Imaging Acquisitions in the Liver and Pancreas. Invest Radiol. 2016;51: 211–220. doi:10.1097/RLI.0000000000000225
