# Supplementary material for: Comparison of six fit algorithms for the intra-voxel incoherent motion model of diffusion-weighted magnetic resonance imaging data of pancreatic cancer patients
Source: PLoS One. 2018 Apr 4;13(4):e0194590. doi: 10.1371/journal.pone.0194590 (PMC5884505; doi:10.1371/journal.pone.0194590)
Supplement: S3 File — (PDF) [file pone.0194590.s003.pdf]

Supporting information III

Table A. Differentiation between tumor and pancreatic tissue.

	Tumour tissue			Normal-appearing tissue		
	D (10^{-3} mm^2/s)	f (%)	D* (10^{-3} mm^2/s)	D (10^{-3} mm^2/s)	f (%)	D* (10^{-3} mm^2/s)
**IVIM-Bayesian-log**	1.36 ± 0.13	7.56 ± 2.09	208.2 ± 106.9	1.43 ± 0.12	8.89 ± 1.49	246.3 ± 111.1
**IVIM-Bayesian-lin**	1.41 ± 0.14	2.56 ± 0.81	83.5 ± 10.2	1.46 ± 0.12	6.38 ± 1.10	101.2 ± 13.4
**IVIM-free**	1.38 ± 0.14	4.98 ± 1.04	56.9 ± 16.6	1.40 ± 0.12	8.22 ± 1.09	93.6 ± 21.3
**IVIM-adaptive**	1.41 ± 0.14	3.94 ± 1.04	58.0 ± 17.4	1.45 ± 0.11	7.31 ± 1.04	98.1 ± 21.3
**IVIM-MLE**	1.43 ± 0.15	4.42 ± 0.99	61.1 ± 17.1	1.49 ± 0.13	7.57 ± 1.01	99.2 ± 21.4
**IVIM-fixed**	1.50 ± 0.15	2.60 ± 0.75	70[a]	1.51 ± 0.12	6.12 ± 1.03	70[a]

Mean value ± standard error (1.96 × SD/√n; n = 14 for tumour tissue, n = 12 for normal-appearing tissue)
[a]D* was fixed to 70 in IVIM-fixed.

Table B. Intra-session wCV.

	Intra-session wCV		
**Fit algorithms**	D	f	D*
**IVIM-Bayesian-log**	9.8	42.7	112.0
**IVIM-Bayesian-lin**	5.8	27.7	26.3
**IVIM-free**	6.3	32.9	32.7
**IVIM-adaptive**	5.3	33.9	39.3
**IVIM-MLE**	5.0	38.7	41.8
**IVIM-fixed**	5.0	23.8	

wCV= between-subject coefficient of variation
